# 

**Published:** 1911-02

**Authors:** 


					﻿PUBLISHED MONTHLY.
SUBSCRIPTION Sl.Of
ATLANTA
JOURNAL-RECORD’
OF MEDICINE"
( Atlanta Medical and Surgical Journal, Established 1855. )	Vfl«r
urtfior to|an<| Southern Medical Record, Established 1870.	) 00111 Tud^
Vol. LVI.
Atlanta, Ga., February, 1911.
No. 11
				

